# Templated electrodeposition as a scalable and surfactant-free approach to the synthesis of Au nanoparticles with tunable aspect ratios

**DOI:** 10.1039/d2na00188h

**Published:** 2022-05-09

**Authors:** Giuseppe Abbondanza, Alfred Larsson, Weronica Linpé, Crispin Hetherington, Francesco Carlá, Edvin Lundgren, Gary S. Harlow

**Affiliations:** Division of Synchrotron Radiation Research, Lund University 221 00 Lund Sweden giuseppe.abbondanza@sljus.lu.se; NanoLund, Lund University 221 00 Lund Sweden; nCHREM, Lund University 221 00 Lund Sweden; Diamond Light Source Didcot OX11 0DE UK; Materials Science and Applied Mathematics, Malmö University 20506 Malmö Sweden gary.harlow@mau.se; MAX IV Laboratory, Lund University Fotongatan 2 224 84 Lund Sweden

## Abstract

A high-throughput method for the fabrication of ordered arrays of Au nanoparticles is presented. It is based on pulsed electrodeposition into porous anodic alumina templates. In contrast to many synthesis routes, it is cyanide-free, prior separation of the alumina template from the aluminium substrate is not required, and the use of contaminating surfactants/capping agents often found in colloidal synthesis is avoided. The aspect ratio of the nanoparticles can also be tuned by selecting an appropriate electrodeposition time. We show how to fabricate arrays of nanoparticles, both with branched bases and with hemispherical bases. Furthermore, we compare the different morphologies produced with electron microscopies and grazing-incidence synchrotron X-ray diffraction. We find the nanoparticles are polycrystalline in nature and are compressively strained perpendicular to the direction of growth, and expansively strained along the direction of growth. We discuss how this can produce dislocations and twinning defects that could be beneficial for catalysis.

## Abstract 2

We synthesised ordered arrays of low-dimensional Au with controllable aspect ratio and morphology. The surfactant-free fabrication, the size-dependent strain state and the twinning defects could be beneficial for catalysis.

## Graphical abstract

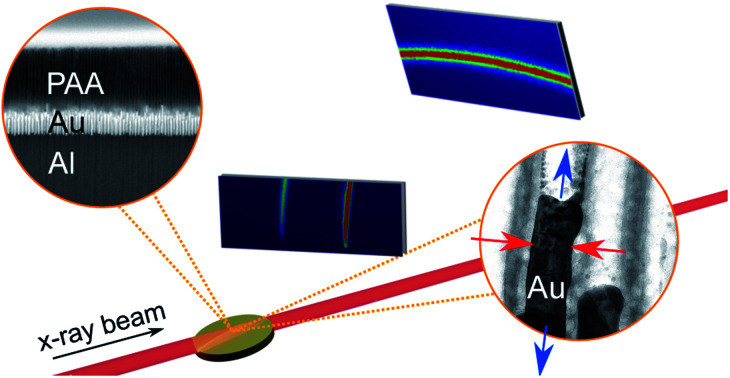

## 1 Introduction

Gold nanoparticles and nanowires have attracted considerable attention in the fields of optoelectronics,^1,2^ catalysis,^3,4^ plasmonics,^5,6^ biomedicine^7,8^ and sensors.^9,10^ Amongst techniques for the synthesis of gold nanoparticles we find thermal evaporation,^11^ aerosol,^12^ spark discharge,^13^ photochemistry^14^ and electrochemical deposition.^15,16^ An attractive and cost-effective approach is electrochemical deposition, which can be combined with template-assisted-growth to produce ordered arrays of nanoparticles with well-defined sizes and shapes.^17^ The template method is an attractive alternative to expensive top-down methods for the fabrication of ordered arrays of one-dimensional materials such as electron beam lithography.^18,19^ When gold nanostructures are arranged in ordered arrays, they find applications in the fabrication of devices,^20^ in microelectrode ensembles^21^ and in nanoscale biosensors.^22^ In addition, thanks to their interesting optical behaviour they are used in the enhancement of the electric field in Raman scattering experiments^23^ and in light harvesting devices.^24^ A promising template for this purpose is porous anodic alumina (PAA) because it has a self-organized long-range order that occurs spontaneously in the anodization of aluminium and is therefore suitable for large-scale production.^25–27^

Electrodeposition in PAA allows accurate control over the aspect ratio of the nanostructures due to two fundamental properties: (i) the deposited nanostructures adopt the shape of the pores. This means that since the pore diameter can be selected by an appropriate choice of electrolyte and electric potential during the anodization, the diameter of the nanostructures can be tuned. (ii) The amount of electrodeposited metal is proportional to the quantity of charge that passed through the working electrode. Since the growth is confined in the horizontal plane, this implies that it is possible to select the height of the nanostructures by controlling the deposition current density (or, alternatively, the electric potential) and the deposition time. Selecting the aspect ratio of noble metal nanorods enables control over the plasmonic and optical properties of the material. For instance, Gans theory predicts that the absorbance peak of Au nanorods increases linearly with the aspect ratio.^28–31^

When a PAA template is fabricated *via* anodization there is an insulating compact oxide barrier layer between the aluminium substrate and the pore bottoms that makes direct electrodeposition difficult.^32^ The homogeneity and success of any electrodeposition into PAA can depend on the thickness (*i.e.* electrical resistance) of the barrier layer, therefore several techniques have been developed to make the barrier layer thinner or to remove it. For example, the alumina template can be detached from the substrate, by a chemical etch of the barrier layer and then one side of the PAA can be coated by a metallic thin film. Metals can then be directly electrodeposited on to the conductive thin-film now at the pore bottoms. This method has proved useful in the fabrication of Au^33–35^ and Cu^36^ and more recently in the synthesis of hierarchically ordered arrays of Ag-nanorod bundles,^37^ flower-like Ag particles^38^ and nanomaterials with magnetic behaviour like Ni,^39^ Co,^40,41^ multi-segmented Fe/Cu^42^ and Fe–Ni–Co alloys.^43^ However, this procedure increases the number of steps required as well as the difficulty of the synthesis; since metal evaporators and the handling of the brittle and thin oxide membrane are needed. In other methods, the thickness of the barrier layer can be reduced significantly through either pore widening (PW) or barrier layer thinning (BLT). PW consists of an isotropic wet chemical etch which causes the gradual and controlled dissolution of the PAA, leading both to an increase of the pore diameter and to the partial dissolution of the barrier layer.^44,45^ The etching agent is usually phosphoric acid (H_3_PO_4_) and the rate can be controlled by tuning the acid concentration, solution temperature and etch duration. The BLT process, instead, involves progressively reducing the anodizing voltage towards the end of the fabrication of the PAA template. This leads to the formation of pore branches in the barrier layer and affects the morphology of the deposited metal.^32,46,47^ Some template preparation methods found in literature^23,32^ involve a combination of PW and BLT, while we used either PW or BLT and compared the morphology, the diameter and the size-dependent strain of the nanostructures deposited in the template.

In addition, the electrodeposition is affected by the electrolyte composition. Common Au electrodeposition baths are often acidic, but acidic or alkaline electrolytes can dissolve the PAA template during the electrochemical growth.^48^ PAA is reported to dissolve at pH values outside the 4–6 range.^49^ Cyanide-based Au baths with neutral pH values can be used^50^ but one would like to avoid this for environmental and health reasons. The waveform of the applied potential also influences the quality of the deposited metal. Pulse electrodeposition (PED) is reported to produce metallic nanostructures with superior quality compared to dc and ac methods, in terms of crystallinity and pore filling.^32,48^

In this work, we propose a synthesis route for the fabrication of ordered Au nanostructures, that can be used for both PW and BLT approaches. Rather than using cyanide-containing molecules to achieve pH neutrality, we used a greener electrolyte solution of HAuCl_4_ and H_2_SO_4_ in a neutral phosphate buffer solution. The neutral buffer not only counters the acidic action of HAuCl_4_ and H_2_SO_4_, but also neutralizes the H^+^ ions produced at the working electrode during the positive voltage pulses, thus avoiding damage and dissolution of the template. In contrast with some colloidal fabrication methods, our electrochemical synthesis does not require surfactants or capping agents to stabilize the size and the morphology of the nanoparticles. This approach is advantageous in fields such as catalysis, since surfactants and capping agents obstruct the reactive surface sites of precious metal nanoparticles hindering any catalytic reaction.^51–55^ Compared to the colloidal synthesis, the template method has the only limitation of a lower yield but it is scalable and it leads to ordered arrays of nanoparticles with controllable inter-pore distance.^56^ We compared the properties and the quality of the different Au nanostructures by means of Focused Ion Beam Scanning Electron Microscopy (FIB-SEM), Transmission Electron Microscopy (TEM), Energy Dispersive X-ray Spectroscopy (EDS), Selected Area Electron Diffraction (SAED) and Grazing-Incidence X-ray Diffraction (GIXRD).

## 2 Experimental

### 2.1 Template preparation

**Fig. 1:**
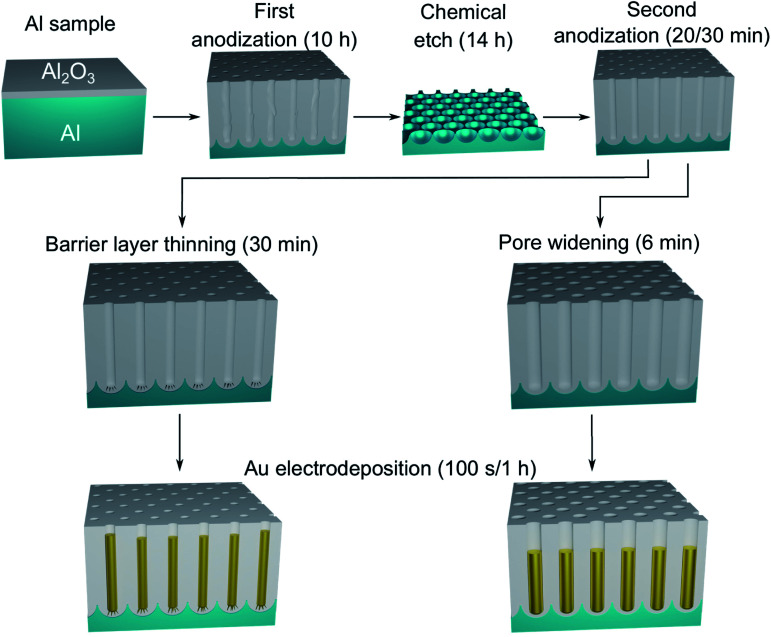
Schematic diagram of the steps involved in the fabrication of the Au nanostructures in PAA. After the two-step anodization process, the PAA template is treated by either BLT or PW. The resulting nanostructures will be either branched or round-bottomed, respectively.

**Fig. 2:**
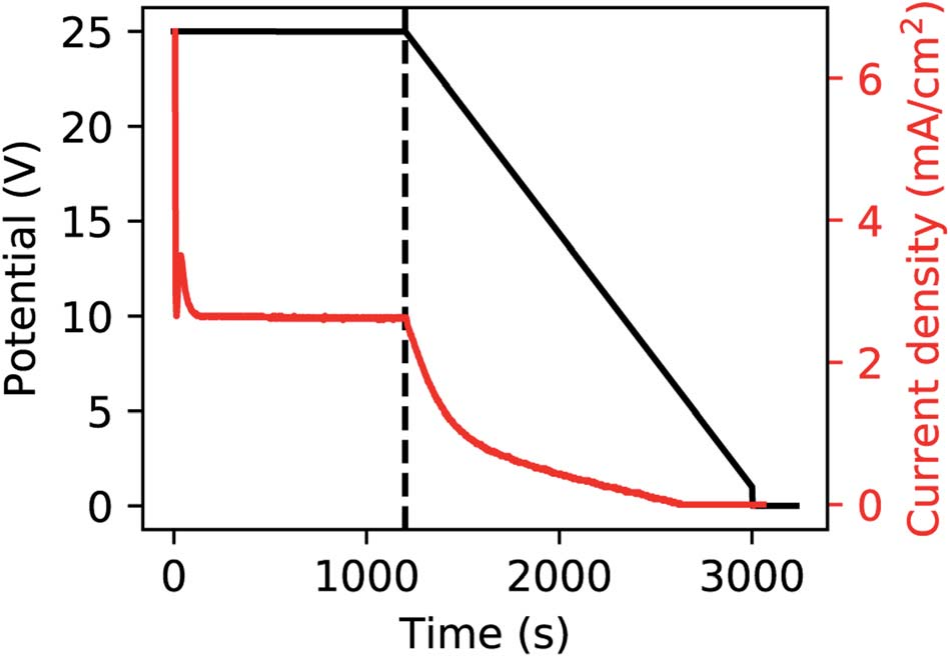
Anodization potential (black) and current (red). The dashed line represents the start of the BLT.

A polished aluminium foil (Goodfellow, 99.999% Al, 50 mm × 50 mm × 0.5 mm) was cut into smaller squared foils (7 mm × 7 mm) and ultra-sonicated in acetone, ethanol and ultrapure H_2_O. All electrolyte solutions in this work were prepared using reagent grade chemicals (Sigma-Aldrich) and ultra-pure water (Millipore, resistivity = 18.2 MΩ cm). The foils were used as substrates for the growth of the PAA templates in a two-step anodization process.^26,57,58^ The Al foils were first anodized in 0.3 M H_2_SO_4_ at 25 V for 10 h while stirring and keeping a constant temperature of 0 °C by using a refrigerated oil bath circulating through a jacketed electrochemical cell. A Kikusui PBZ-20-20 programmable bi-polar power supply was used for the anodization. The resulting PAA was stripped from the Al matrix using a mixture solution of 0.185 M H_2_CrO_4_ and 0.5 M H_3_PO_4_ for 14 h at room temperature, which leads to an Al substrate patterned with nano-concaves. A greener chromic-acid-free fabrication could be achieved by using substrates pre-patterned with an imprint mould.^59^ The second anodization was performed in the same conditions of electrolyte, potential and temperature as the first anodization, but for a duration of either 30 min or 20 min. The steps of this fabrication process are illustrated in Fig. 1. A PW step was performed on the samples anodized for 30 min by immersion in a solution of H_3_PO_4_ 5 wt% at the temperature of 30 °C for 6 min, while the solution was stirred to reduce convection. The samples anodized for 20 min were treated by means of BLT, performed by decreasing the anodization potential from 25 V to 1 V over 30 min, immediately after the second anodization step as shown in Fig. 2.

### 2.2 Au electrodeposition

**Fig. 3:**
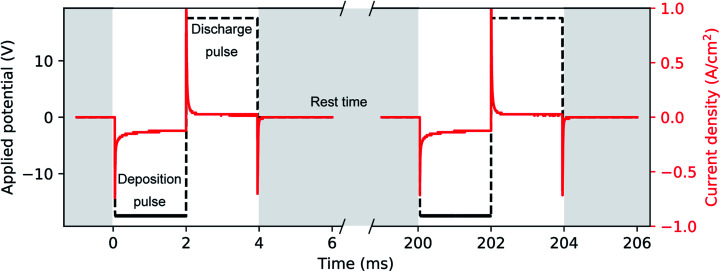
Typical transients of applied voltage and geometrical current density during Au deposition into PAA. A cathodic square deposition pulse (2 ms) is followed by an anodic pulse of same duration and voltage in absolute value (*i.e.*, 17.5 V). The pulses are repeated after a rest time of 196 ms to restore the HAuCl_4_ concentration in the pores.

A phosphate buffer solution was prepared by slowly mixing and stirring diluted H_3_PO_4_ and KOH to a final concentration of 1 M and 1.95 M, respectively. This solution not only acts as a conductive supporting electrolyte for the electrochemical deposition, but it has a constant and nearly neutral pH of 7.5. The electrodeposition bath was completed with the addition of 10 mM H_2_SO_4_ and 10 mM HAuCl_4_, after which we measured a pH of 7.2. The role of H_2_SO_4_ is to increase the conductivity of the solution and to enhance the stability of Au(iii) species in solution.^60^ Unlike the acidic (pH = 4.5) Watts bath, which is common in Ni electrodeposition^32^ and has a high concentration (2 M) of Ni(ii) in solution, our Au bath has a neutral pH and a relatively smaller concentration (10 mM) of Au(iii). PED was conducted with a two-electrode setup where the aluminium substrate covered with the PAA template acted as the working electrode and a Pt rod was the counter electrode. The electrodeposition bath was at room temperature and continually stirred. During PED a 2 ms square cathodic potential pulse of −17.5 V was applied to the working electrode, during which Au is electrodeposited. This deposition pulse is followed by a square anodic pulse of 17.5 V also of 2 ms, to discharge the capacitance of the barrier layer. A rest period of 196 ms is then used to allow the concentration of the metal ions (*i.e.*, AuCl^−^_4_) inside the pores to replenish. An example of the applied potential is shown in Fig. 3. The potential pulses were applied by using the Kikusui PBZ-20-20 programmable power supply, used above for the anodization. The benefits of this electrodeposition method have been extensively studied.^32,48^ The electrodeposition was monitored by recording the applied voltage and the resulting current with a two-channel digital oscilloscope (PicoScope 3000). The voltage across a shunt resistor (25 Ω) in series with the electrochemical cell was measured and the current was calculated using Ohm's law. Based on the duration of the electrodeposition and on the template preparation, it was possible to fabricate nanorods, branched nanorods or spheroidal nanoparticles. Fig. 1 summarizes the experimental conditions that lead to a particular morphology of the nanostructures. After the electrodeposition, the samples were rinsed in ultra-pure water.

### 2.3 Characterization

#### 2.3.1 FIB-SEM

A Nova NanoLab 600 dual beam FIB-SEM (FEI Company) was used to mill away material from the PAA samples and expose the embedded Au nanostructure to a clear cross-sectional view. Using FIB-assisted lithography a protective Pt strip (2 × 20 μm) was deposited on the sample. The milling was conducted using the FIB with an accelerating potential of 30 kV. SEM imaging was conducted either using a potential of 5 kV and secondary electron detection or 10 kV and backscatter electron detection. A thin lamella was extracted by milling material away with the FIB-SEM from a sample containing Au nanowires. The lamella was extracted with an Omniprobe™ from the porous alumina matrix and welded with FIB-assisted Pt deposition onto a TEM post.

#### 2.3.2 TEM

TEM, EDS and SAED measurements were performed at the National Center for High Resolution Electron Microscopy in Lund (Sweden). In preparation for the measurements, the Au nanoparticles were released from the PAA template by dissolution in 1 M NaOH at room temperature. After allowing the nanostructures to precipitate by letting the solution rest overnight, the mixture was rinsed generously, removing the supernatant and replacing it with ultra-pure H_2_O. This rinsing process was iterated 10 times and the last time the solvent was replaced with ethanol. To disperse the nanostructures, the solution was sonicated for 10 minutes. A solution volume of 20 μL was then pipetted onto a TEM grid. A JEM3000F TEM operated at 300 kV was used to analyse the released Au nanoparticles, while a JEM-2200FS TEM was used to image the nanostructures embedded in the PAA lamella.

#### 2.3.3 Synchrotron GIXRD

**Fig. 4:**
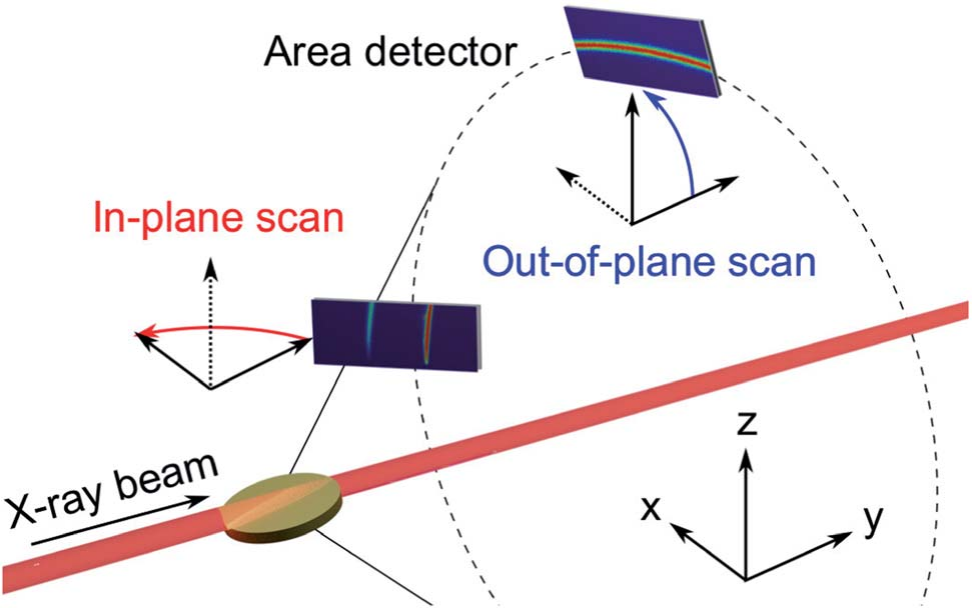
GIXRD geometries: in the in-plane scans (red) the detector rotates about the *z*-axis, while the out-of-plane scans (blue) the detector rotates about the *x*-axis.

A sample of Au electrodeposited in a pore-widened PAA template was analysed by means of XRD at beamline I07 (Diamond Light Source, UK), which is dedicated to X-ray diffraction at surfaces and interfaces.^61^ The sample was mounted on a Huber (2 + 3)-type diffractometer^62^ equipped with an Excalibur detector.^63^ The beam had a size of 100 × 300 μm (vertical × horizontal) and an energy of 20 keV. The diffractometer was calibrated using a NIST LaB6 standard reference material. To investigate the possible presence of texture and preferential orientations of the nanowires, we recorded X-ray diffraction in the two grazing-incidence geometries illustrated in Fig. 4: in the horizontal (*x*,*y*)-plane and in the vertical (*y*,*z*)-plane. The detector was scanned in steps of 0.1° while the sample was kept at a grazing-incidence angle of 0.5°. In the in-plane geometry, the scattering vector always lies on the horizontal (*x*,*y*) plane, *i.e.*, in the direction of confinement. On the other hand, when the Bragg condition is met in an out-of-plane scan, the scattering vector forms an angle of *θ*_*hkl*_ − *α* with the surface normal, where *θ*_*hkl*_ is the diffraction angle and *α* is the incidence angle. The two-dimensional data collected while scanning the area detector were converted to azimuthal regroupings using the angle calculations described in previous research.^64^

## 3 Results

**Fig. 5:**
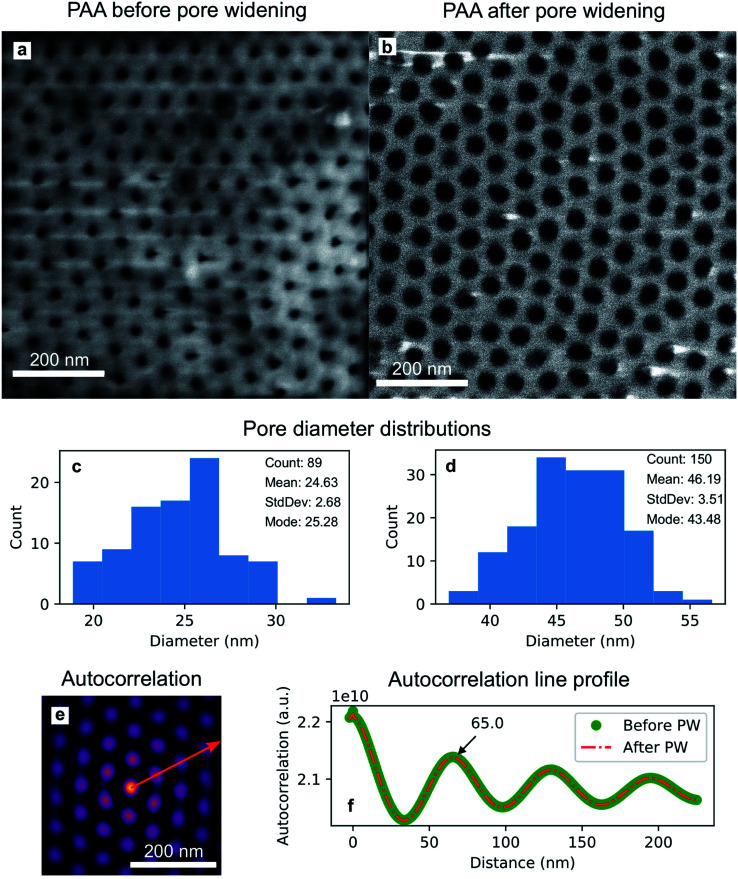
SEM micrographs of a PAA template before (a) and after (b) the PW, with their respective distributions of pore diameter, (c) and (d). Autocorrelation function (e) of the image shown in (b). Line profile (f) of the autocorrelation function, plotted along the red arrow in (e), for before and after the pore widening.

**Fig. 6:**
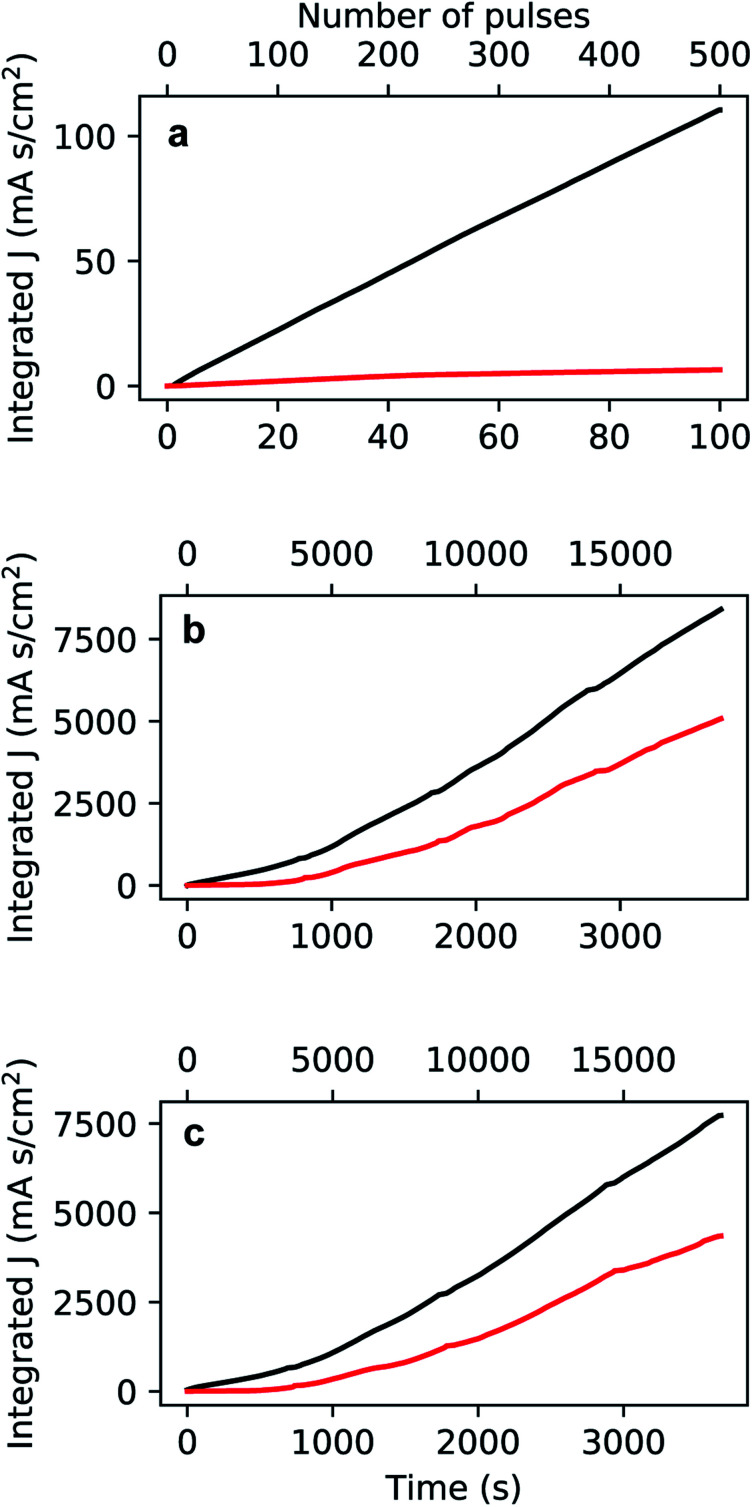
Integrated current density recorded during the growth of Au nanoparticles (a), high-aspect ratio nanoparticles with round bases (b), and high-aspect ratio branched nanoparticles (c). The cathodic (black) and anodic (red) curves are reported in absolute value against deposition time and number of pulses.

The isotropic action of PW leads to changes in the PAA template morphology. In Fig. 5, two SEM micrographs of a PAA template are compared before (a) and after (b) PW. The images have been processed with the Fiji ImageJ software^65^ to obtain the distribution of pore diameters [(c) and (d)]. The pore diameters increased by approximately 22 nm after PW, leading to a thinning of the barrier layer, while the interpore distance is unchanged. The autocorrelation function shown in Fig. 5e is an expression of the hexagonally structured order of the pores and it was calculated using the software Fiji ImageJ. If we express an image as *f*(*u*,*v*), where *u* and *v* are the pixel coordinates, the autocorrelation product can be expressed as1
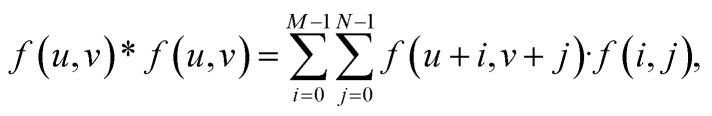
where * denotes the correlation product and the summations are extended over the *i*-th and *j*-th pixel of the N rows and M columns of the image. This equation is a useful tool for finding repeating patterns like the hexagonally ordered pores fabricated in this work. A line connecting the centre of the auto-correlation pattern to the nearest peak was drawn in ImageJ. This line is shown in red in Fig. 5e. The length of the line was extended to cover more peaks than the nearest one only. The auto-correlation profile was extracted and plotted in Fig. 5f. This protocol was followed for both the SEM images in Fig. 5a and b, and therefore the auto-correlation lines from before and after pore widening are shown in Fig. 5f. The first maximum in the line profiles plotted in Fig. 5f represents the average interpore distance and is located at 65.0 nm, in good agreement with values found in literature ranging from 62.5 nm (ref. 66) to 68.6 nm (ref. 67). As the PW only affects the pore diameter, the interpore distance is unaltered. Fig. 6 reports the integrated current density flowing through the electrochemical cell during the Au electrodeposition. It shows the total charge *Q*, normalized by geometric surface area, during the cathodic (black) and the anodic (red) pulses from the beginning of the deposition to any instance *t*, as expressed by the following equation:2
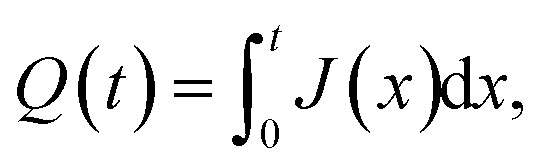
where *J* is the current density and *x* is a dummy integration variable. Fig. 6a shows the integrated current for the electrodeposition of Au spheroidal nanoparticles, while (b) and (c) show the integrated currents for Au nanorods and branched nanorods, respectively.

**Fig. 7:**
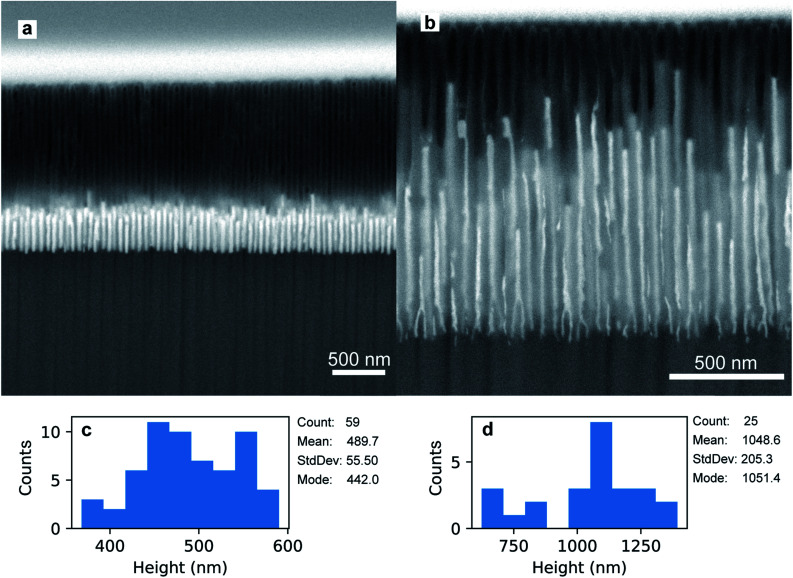
FIB-SEM micrographs of high-aspect ratio Au nanoparticles embedded in PAA treated by means of PW (a) and BLT (b). In both images, the tilt angle is 52°. The distributions in (c) and in (d) refer to the height of the nanoparticles in (a) and (b), respectively.

The preparation of the PAA template has an impact on the morphology and the size of the electrodeposited Au nanoparticles. Fig. 7a shows a FIB-SEM micrograph of the high-aspect ratio Au nanoparticles embedded in a PAA template treated by PW. These nanoparticles have round tips and appear to have a homogeneous average size of 489.7 nm (aspect ratio ≈ 11). The height distribution in Fig. 7c has a standard deviation of 55.5 nm (11%). On the other hand, the branched-base high-aspect ratio nanoparticles in Fig. 7b, grown in a template prepared by means of BLT, appear to be less homogeneous in height, with a standard deviation of 205.3 nm (20%) [see Fig. 7d].

**Fig. 8:**
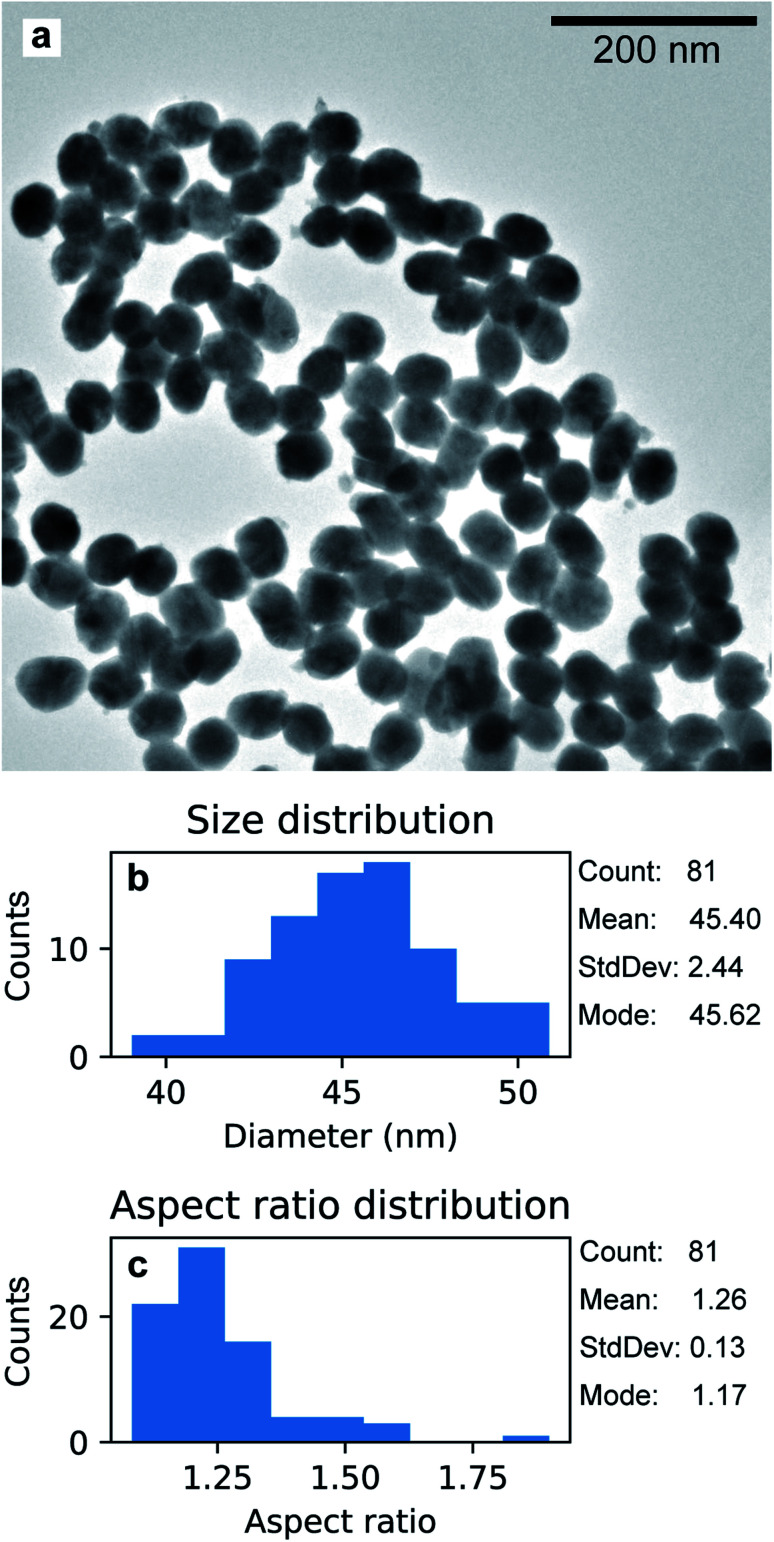
TEM micrograph of Au nanoparticles grown with electrodeposition time 100 s, after being released from the PAA template (a). Distribution of size (b) and aspect ratio (c).

**Fig. 9:**
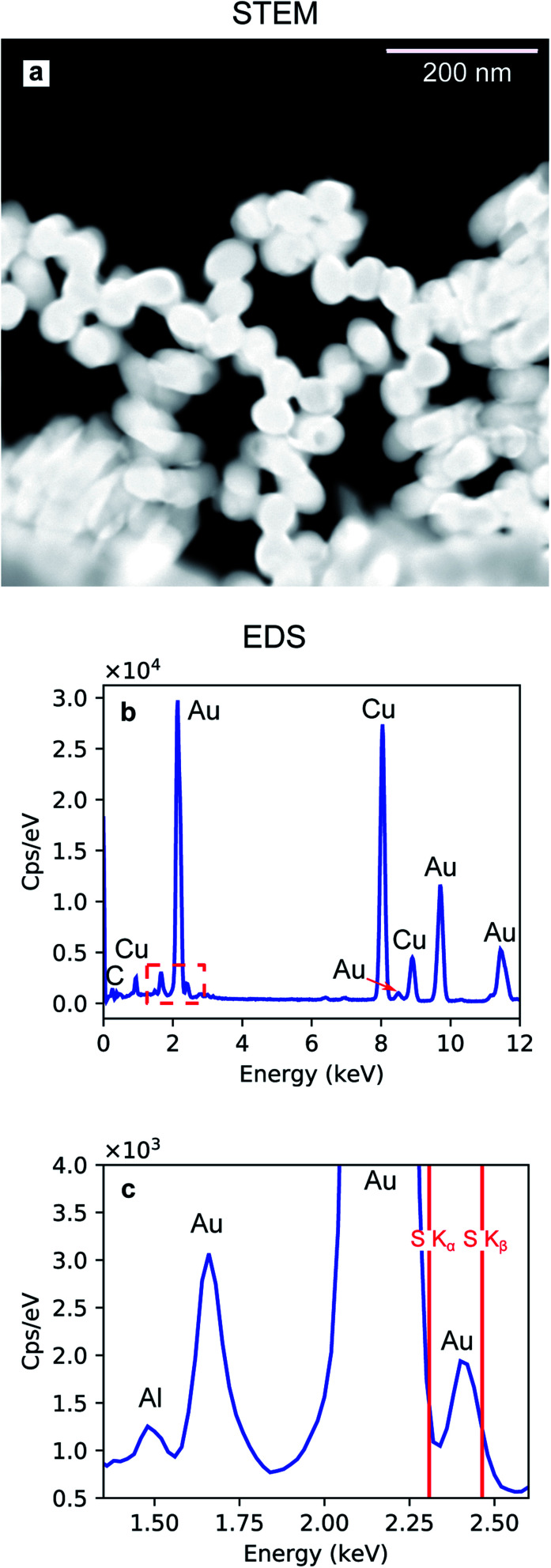
Dark-field STEM image of Au nanoparticles released from the PAA template (a). EDS spectrum (b) recorded from the area shown in (a). Magnified section of the EDS spectrum including the characteristic Al K*α* (c).

The Au nanoparticles in Fig. 8a were prepared keeping the same conditions as those for the high-aspect ratio particles and changing only the deposition time (100 s). Here, the synthesized nanostructures have a size distribution of 45.4 nm with a standard deviation of 2.44 nm (5%), as shown in Fig. 8b. These nanoparticles were released from the template upon selective dissolution of Al_2_O_3_ and dispersed on a TEM grid as described in Section 2.3.2. To obtain information on the composition of the deposited Au nanoparticles, EDS was performed. Fig. 9b shows the EDS spectrum from the selected area in Fig. 9a, which shows the same Au nanoparticles as those in Fig. 8a, *i.e.*, released from the PAA template. The spectrum features the characteristic emission peaks of Au and those originating from the TEM grid, *i.e.*, C and Cu. Fig. 9c show a close up of the boxed area in Fig. 9b around the Au M line. Furthermore, the absence of S K*α* and K*β* emission lines suggests that the nanoparticles are not contaminated by sulphur, which is present in both the anodization and in the electrodeposition solutions. The Al peak at 1.486 keV is attributable to residuals of undissolved alumina on the Au nanoparticles.

**Fig. 10:**
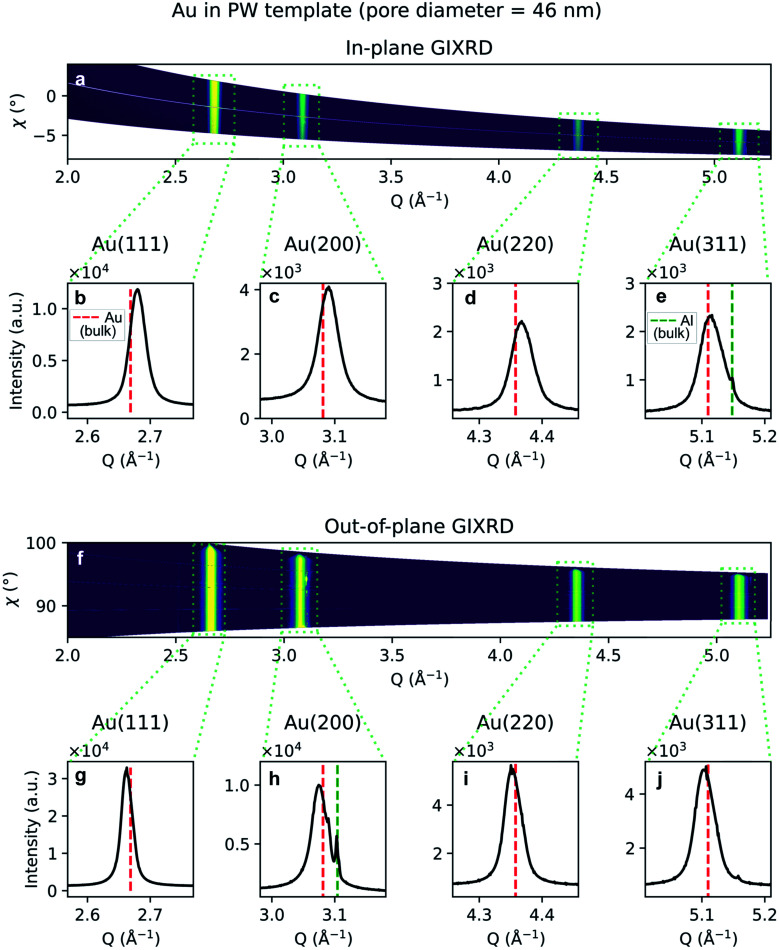
GIXRD data from Au nanoparticles in a template treated by PW. (a) Two-dimensional regrouping of the images from the in-plane scan. (b–e) Powder diffraction peaks integrated from the integrated boxes in the in-plane scan data regrouping. (f) Two-dimensional regrouping of the images from the out-of-plane scan. (g–j) Powder diffraction peaks from the integrated boxes in the out-of-plane scan data regrouping. The theoretical positions of bulk Au and Al are marked by a red or green dashed line, respectively.

Due to the penetrative nature of hard x-rays, GIXRD can be used to investigate the nanoparticles while they are embedded in PAA. The GIXRD data in Fig. 10 shows diffraction peaks arising from the face-centred-cubic (fcc) structure of Au electrodeposited in a template treated by means of PW. In the two-dimensional plots in Fig. 10a and f, the diffraction intensity from the in-plane and the out-of-plane scans are plotted as a function of both the magnitude of the scattering vector *Q* and the azimuthal angle *χ*. The boxed areas in the two-dimensional regroupings were integrated and plotted in Fig. 10b–e and g–j, where the theoretical positions of bulk Au peaks are reported by a red dashed line. In Fig. 10e and h some weak peaks appear which match the theoretical position of the Al(200) and (311) peaks (green dashed line).

**Fig. 11:**
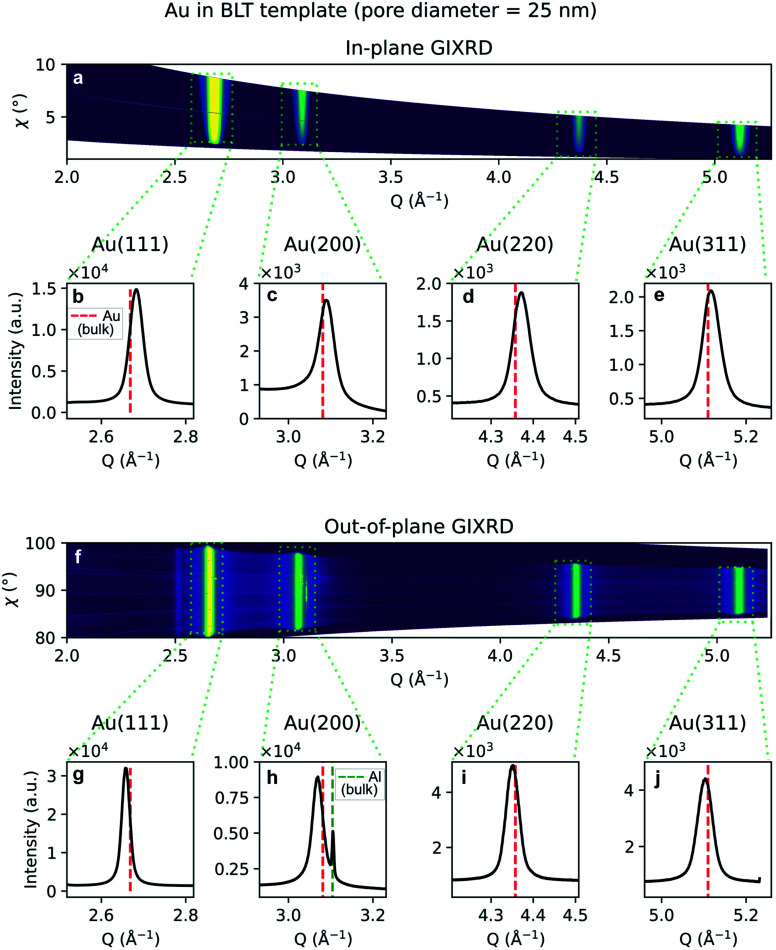
GIXRD data from branched Au nanoparticles in a template treated by BLT. (a) Two-dimensional regrouping of the images from the in-plane scan. (b–e) Powder diffraction peaks integrated from the integrated boxes in the in-plane scan data regrouping. (f) Two-dimensional regrouping of the images from the out-of-plane scan. (g–j) Powder diffraction peaks from the integrated boxes in the out-of-plane scan data regrouping. The theoretical positions of bulk Au and Al are marked by a red or green dashed line, respectively.

Similarly, the GIXRD arising from branched Au nanostructures in a template treated by BLT, is shown in Fig. 11a and f for the in-plane and out-of-plane regroupings, respectively. Fig. 11b–e and g–j represent the one-dimensional integral of the boxed diffraction peaks. In Fig. 11h we also observe an Al(200) peak. In both samples we observe homogeneous and broad diffraction rings arising from the gold.

**Fig. 12:**
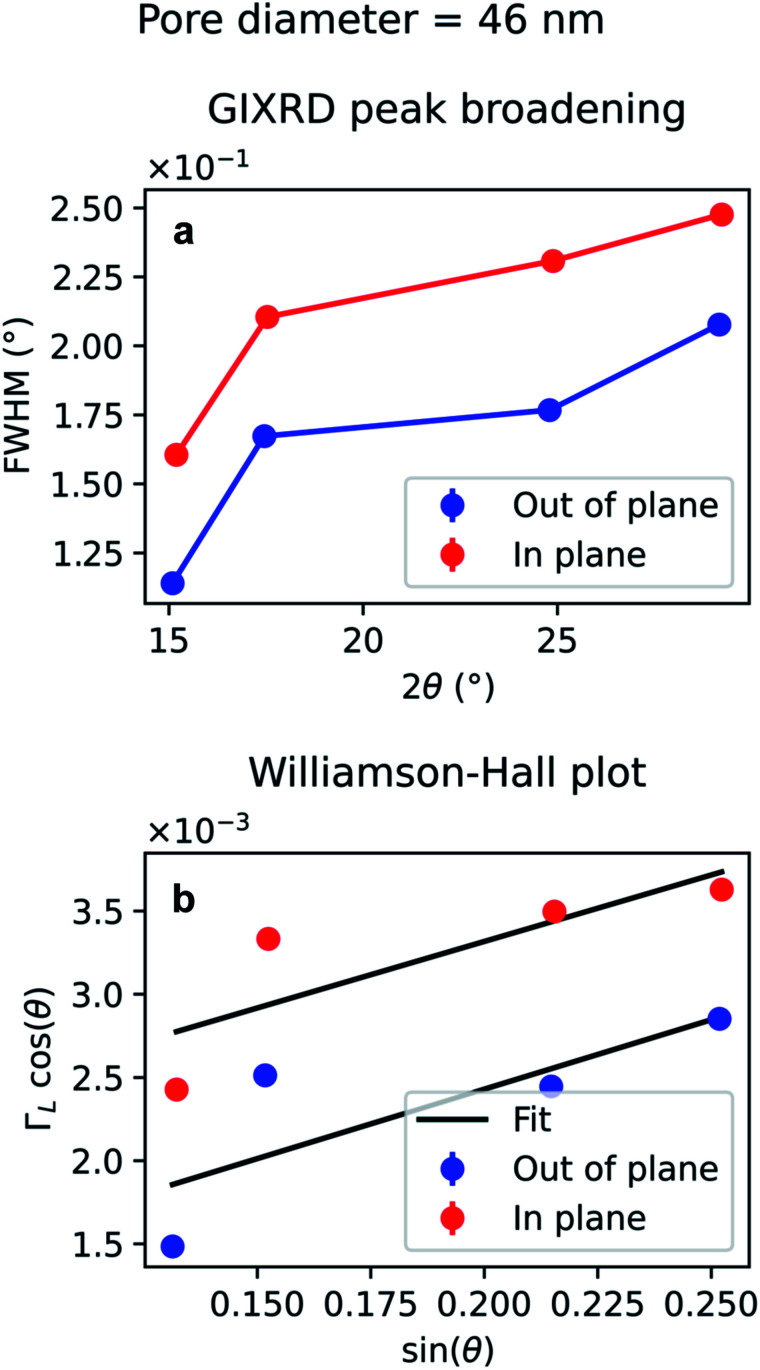
Peak broadening analysis of the GIXRD data from Au nanoparticles in a template treated by PW. (a) Full-width half maximum of the Au peaks as a function of the diffraction angle 2*θ* (b) Williamson–Hall plot of the Lorentzian FWHM, *Γ*_L_, for out-of-plane and in-plane data. The error bars in (b) are too small to be displayed and derive from the uncertainty of the peak fitting to estimate the Lorentzian contribution.

**Fig. 13:**
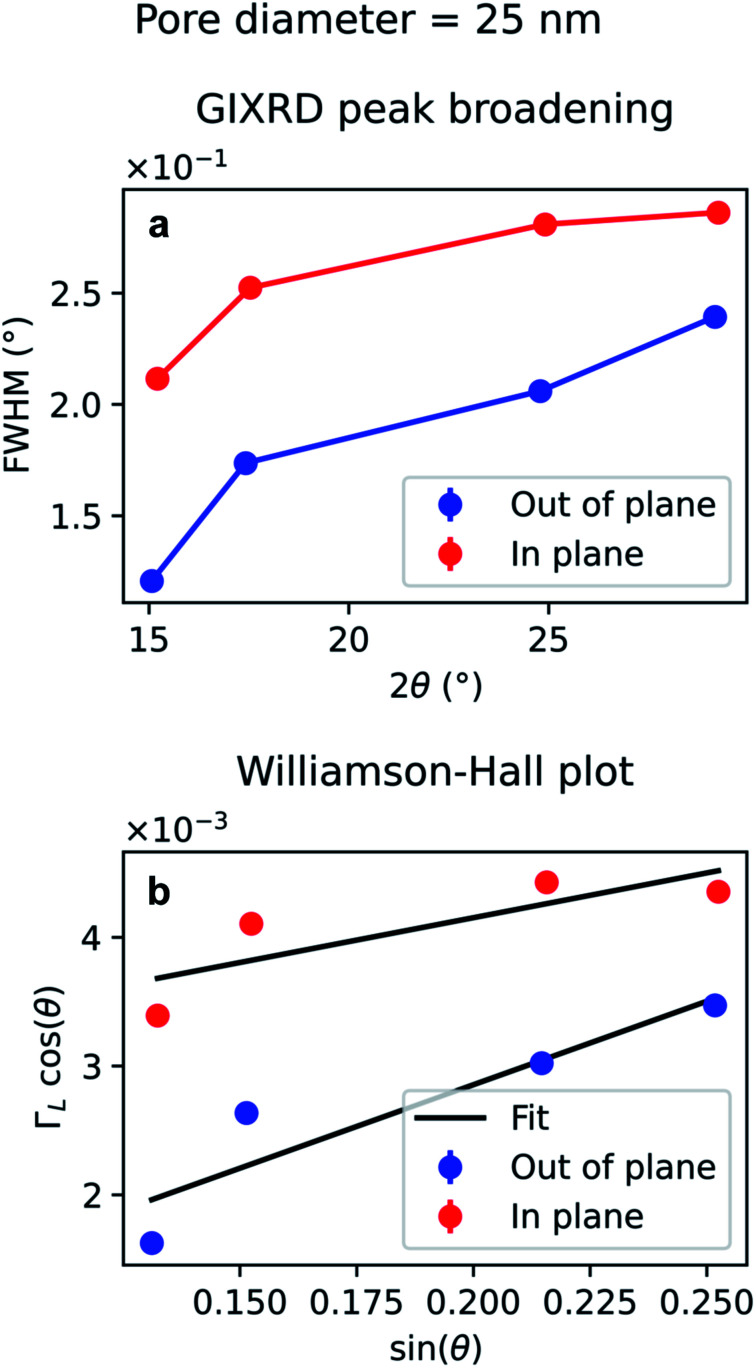
Peak broadening analysis of the GIXRD data from branched Au nanoparticles in a template treated by BLT. (a) Full-width half maximum of the Au peaks as a function of the diffraction angle 2*θ* (b) Williamson–Hall plot of the Lorentzian FWHM, *Γ*_L_, for out-of-plane and in-plane data. The error bars in (b) are too small to be displayed and derive from the uncertainty of the peak fitting to estimate the Lorentzian contribution.

The diffraction peak width contains information on the crystallite size. Since the scattering vector lies in the horizontal plane during the in-plane scans, and in the vertical plane in the out-of-plane scans, it is possible to extract the horizontal and vertical crystallite size. The full-width-half-maximum (FWHM) for both samples and both experimental geometries are shown in Fig. 12a and 13a. In both templates, the FWHM is higher in the in-plane scans than in the out-of-plane scans. This suggests that the vertical grain size is larger than the horizontal one. To verify that, the peaks were fitted with Voigt functions and the Lorentzian component of the FWHM was used to produce Williamson–Hall plots, shown in Fig. 12b and 13b, where the *y*-axis intercept is inversely proportional to crystallite size.^68^ For the nanoparticles in the PW template, the Williamson–Hall analysis revealed a crystallite size of 36.1 ± 4.1 nm horizontally and of 82 ± 17 nm vertically, while the crystallites of the branched nanoparticles in the BLT template have a horizontal size of 22.5 ± 3.8 nm and a vertical size of 237 ± 15 nm.

**Fig. 14:**
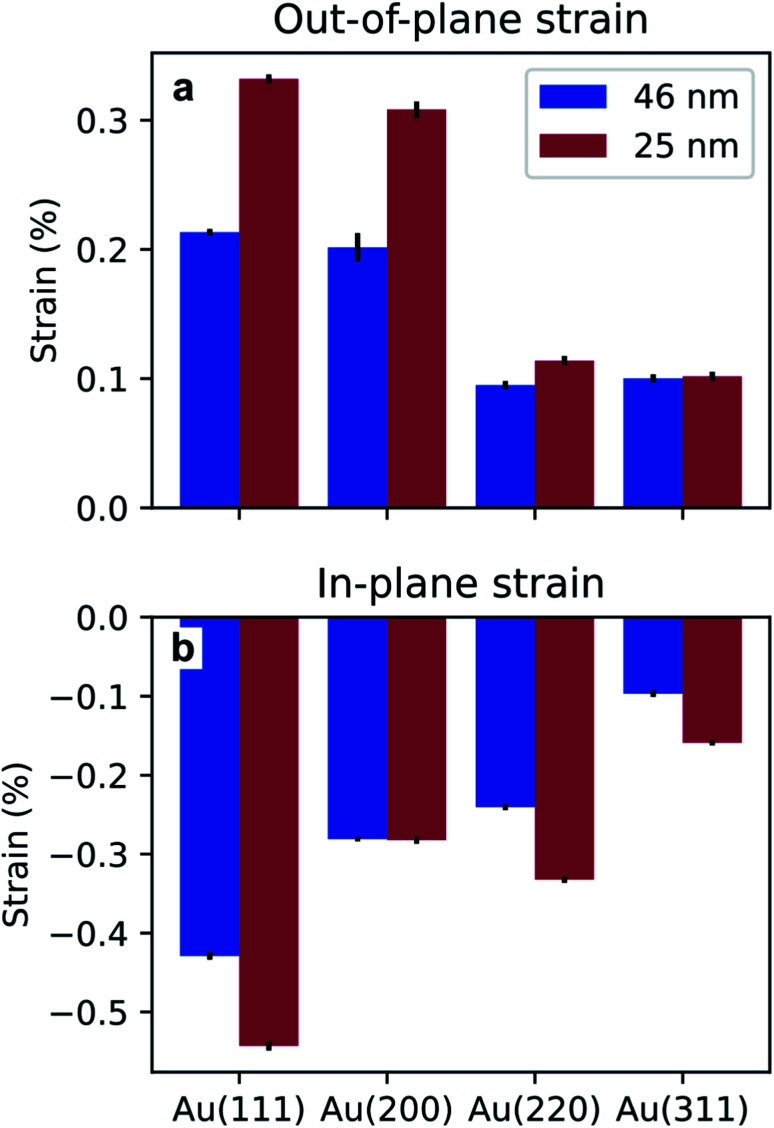
Homogeneous strain extracted from the shift of the peaks in the vertical (a) and horizontal (b) direction, for the two pore sizes investigated in this work.

A shift in peak positions from the theoretical values predicted by Bragg's law was observed. The theoretical bulk position is marked by a dashed line in Fig. 10b–e and g–j as well as in Fig. 11b–e and g–j. The peak shift from the theoretical bulk position is due to the presence of homogeneous strain. Fig. 14 reports the calculated strain for both GIXRD geometries and for both kinds of templates investigated in this work.

**Fig. 15:**
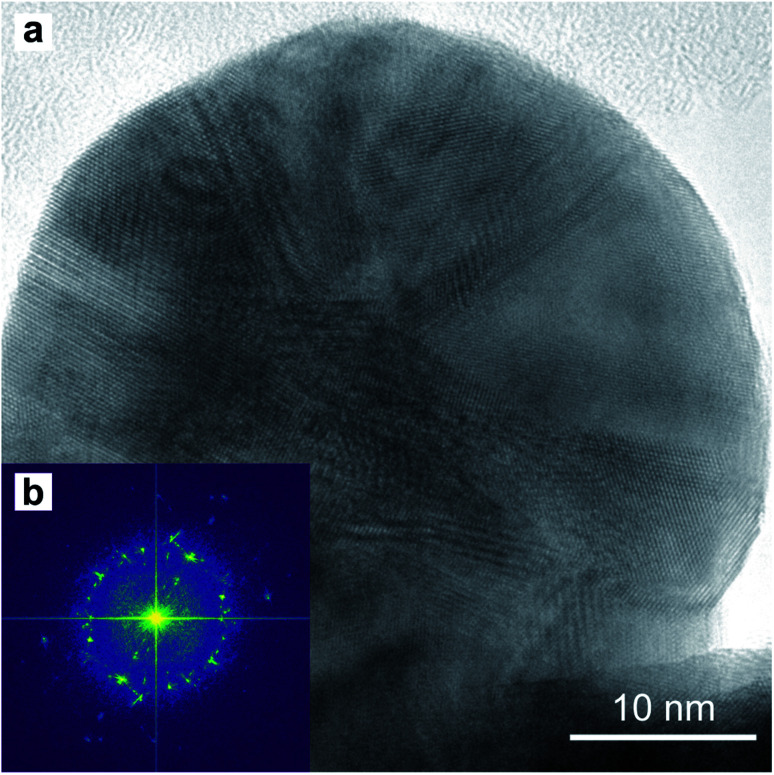
HR-TEM image (a) of a round-based low-aspect ratio Au nanoparticle, released from a pore-widened PAA template. Fast-Fourier Transform (b) of the image in (a).

A TEM image of a low-aspect ratio Au nanoparticle is shown in Fig. 15a after release from the PAA template by dissolution with 1 M NaOH. This and other images of nanoparticles show planar defects which we can conclude are twin planes, and the outline shape has facets and indents where the twin plane meets the particle surface. There are also Moiré-type fringes caused by overlapping crystals at different orientations. The Fourier-transform shown in Fig. 15b is calculated from the image in (a) and indicates a polycrystalline fcc material, while some sub-regions in the image show familiar 110 projections of the Au fcc lattice.

**Fig. 16:**
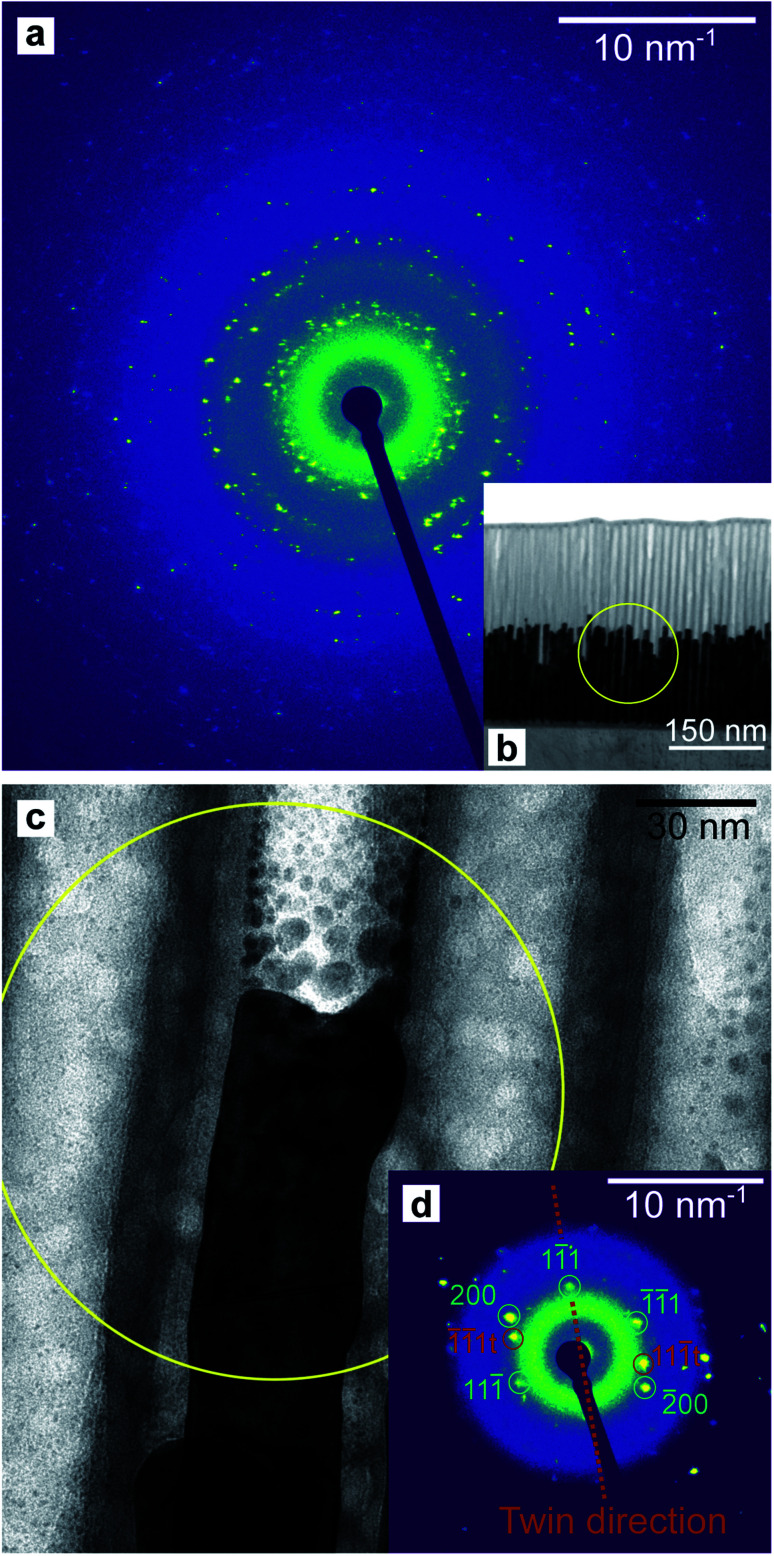
SAED pattern (a) of the Au nanorods embedded in a PAA lamella, arising from the circular region displayed in (b). The pattern in (d) is from a smaller region highlighted in yellow in (c) which included the end of a single Au rod. The circular defects in (c) are holes in the PAA caused by prolonged exposure to the electron beam.

A thin lamella was extracted from a sample of high-aspect ratio Au nanoparticles embedded in a PAA template treated by PW. Fig. 16b shows a TEM image of the lamella and a SAED pattern (a). This pattern was recorded by exposing only the Au nanorods and PAA to the electron beam, thus avoiding contribution from the Al substrate. Fig. 16c shows the tip of an Au nanorod and the selected area for the pattern which is shown in Fig. 16d.

## 4 Discussion

The conditions of anodization and the method employed to thin the barrier layer have an impact on the parameters describing the self-ordered structure of PAA, such as the pore diameter (*D*_p_), the interpore distance (*D*_int_), the barrier layer thickness (*t*_b_), the pore wall thickness (*t*_w_), the pore density (*ρ*_p_) and the porosity (*P*).^69^ The extent of the thinning can be estimated by simple geometric considerations. The interpore distance can be expressed as

3 *D*_int_ = *D*_p_ + 2*t*_w_,

and according to the empirical relation found in literature for templates fabricated in H_2_SO_4_:^70^

4 *t*_b_ = 1.33*t*_w_.

Therefore, the barrier layer thickness can be expressed as

5 *t*_b_ = 0.665(*D*_int_ − *D*_p_).

Using eqn (5), we find a barrier layer of 26.8 nm before PW and 12.5 nm after PW. This calculation works in the assumption that the etching action of the H_3_PO_4_ is isotropic and that it dissolves the PAA walls as much as the barrier layer. The pore density (*ρ*_p_) and the porosity (*P*) of the templates can be expressed as6
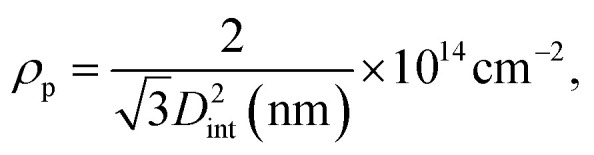
7
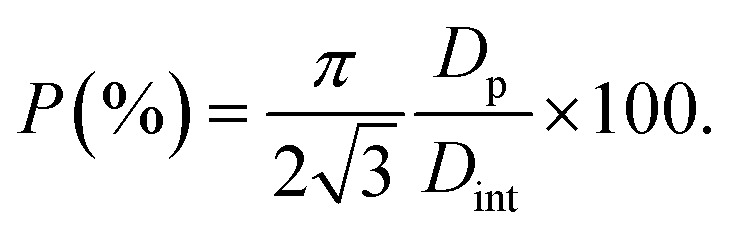


The pore density depends only on the inter-pore distance, which is unaffected by the PW and therefore equals 2.73 × 10^10^ pores per cm^2^ in both before and after PW. The porosity, instead, varies from 34.4% to 64.5%. On the other hand, the BLT leaves the pore diameters intact and the porosity unaffected and acts only on the thickness of the barrier layer. Some empirical relations between the anodizing voltage and tb (*i.e.*, the “anodizing ratio”) have been reported in literature. This ratio was determined to be approximately 1 nm V^−1^ for H_2_SO_4_^71^ in conditions of constant anodizing potential. In our case, the potential is ramped down from 25 V to 1 V, therefore the barrier layer thickness will be in a range between 25 nm to 1 nm.

The current plots in Fig. 6 show a dominance of the cathodic current over the anodic current which means that most of the reactions are driven by a cathodic potential at the working electrode, amongst which is the reduction of Au(iii): AuCl^−^_4_ + 3e^−^ → Au + 4Cl^−^. A linear increase is seen in the integrated cathodic current [Fig. 6a] which suggests the steady-state growth of the Au nanoparticles. On the other hand, in Fig. 6b and c, after approximately 600 s from the start of the deposition, there is a non-linear increase in both the cathodic and anodic currents. This deviation can be possibly explained by (i) an increased reactivity and (ii) an increased capacitance on the interface of the working electrode, due to the presence of the Au nanostructures.

The Au nanorods grown in a PW template [Fig. 7a] have a more homogeneous height compared to those grown in a BLT template [Fig. 7b]. This is evidence that PW leads to a more homogeneous barrier layer. The wide distribution of heights in the BLT sample is probably due to the fact that the barrier layer does not have the same thickness in all the pores. Since the transmittance of a particle through a potential barrier *via* quantum tunnelling varies exponentially with the barrier width,^72^ the probability that an electron tunnels through the alumina during an electrodeposition pulse goes down exponentially with the thickness of the barrier layer. Therefore, even small variations of thickness can have a significant impact on the resulting aspect-ratio of the Au nanoparticles. Although the BLT does not offer as precise control of *t*_b_ over all the pores, the resulting Au nanoparticles feature a higher average length of 1049 nm and a superior aspect ratio of 42. To obtain nanowires with equal length, one could grind or polish the topmost part of the PAA.^33^

The aspect ratio can be controlled by choosing an adequate deposition time. Short deposition times such as 100 s lead to the formation of the spheroidal nanoparticles shown in Fig. 8a. As the mass of deposited material should be proportional to the total charge passing through the working electrode during the cathodic pulses, a shorter deposition time results in shorter nanoparticles (average size of 45.4 nm) featuring a narrow size distribution with a relative standard deviation of 5.3%. Controlling the aspect ratio in such a simple way might be advantageous in the fabrication of noble metal nanostructures for plasmonic and optical applications. The dependence between the absorbance peaks of Au nanoparticles in the UV/visible wavelength range scales linearly with the aspect ratio.^28,30,31^

For both the templates studied in this work, the pore size is comparable with the horizontal crystallite size extrapolated from the Williamson–Hall plots in Fig. 12b and 13b. In fact, the Au nanoparticles have a horizontal crystallite size of 36 ± 4.1 nm in the PW template, which has a pore diameter of 46 nm, while they have a crystallite size of 22 ± 3.8 nm in the BLT template, which has a pore diameter of 25 nm. This means that the horizontal grain size is limited by the size of the pores. On the other hand, the vertical crystallite size of the nanoparticles in the BLT template (237 ± 15 nm) is larger than that for the nanoparticles in the PW template (82 ± 17 nm). This shows how nanoparticles in smaller pores are less fragmented by grain boundaries.

The homogeneity of the powder diffraction rings in Fig. 10a, f and in Fig. 11a, f suggests a lack of texture and the absence of preferred orientations. This evidence is strengthened by the SAED measurement in Fig. 16a, which arises from the array of nanostructures in Fig. 16b. Here, the randomly distributed diffraction intensity along some powder rings expresses the random orientation of the crystallites. The SAED pattern in Fig. 16d is taken from the outlined area in (c). The indexed pattern shows a 011 beam direction through a region of fcc crystal which is twinned, and twin reflections are highlighted in red.

Although some XRD Al peaks are present in Fig. 10e, h and 11h, the EDS elemental analysis did not show any significant contribution from Al. Therefore, we can exclude that the XRD Al signal originates from possible contamination of the nanostructures. The presence of the Al Bragg reflections is rather due to the X-ray penetration into the Al substrate. In fact, at an incidence angle of 0.5° and a beam energy of 20 keV, the penetration depth into an Al_2_O_3_ slab is 10.6 μm. Since the template thickness is only 2 μm, part of the incident X-rays penetrates through the Al substrate and excites some Bragg reflections.

Furthermore, the analysis of the XRD measurements revealed some homogeneous strain of the nanostructures. From Fig. 14, it appears evident that the strain is compressive in the direction of confinement and expansive in the direction of growth. Although the origin of the stress that causes this deformation is unclear, it has been attributed to growth in confined space for the case of Sn nanowires.^46,73^ Furthermore, the strain is more pronounced in the template with the smaller pores, *i.e.*, the BLT template. This is due to a size effect previously reported in literature where the smaller the pores, the higher the growth stress.^74^ The strain for one particular geometry and for one kind of template varies from peak to peak. This might be due to the fact that the Young's modulus depends on the crystallographic orientations.^75^ These observations about homogeneous strain are consistent with previous findings on the electrodeposition of Pd^76,77^ and Sn^46^ in PAA.

The twinning defects on the tip of the nanostructures in Fig. 15a and might be a result of the growth stress in the PAA template. In fact, dislocations and twinning defects are often a consequence of stress accumulation in fcc metals.^78–80^ This might be an advantageous feature since twin boundaries are known to enhance the electrocatalytic activity of fcc metal nanowires.^81–83^ The nanostructures fabricated in this work might then be an attractive alternative to colloidal nanocatalysts. Some colloidal nanocatalysts need a step of surfactant/capping-agent removal to activate the catalytic properties.^84–86^ Since the electrochemical method proposed in this work does not make use of surfactants nor capping agents, this step would not be required. However, one disadvantage of the template method is the lower yield, compared to a typical citrate-stabilized seed-mediated colloidal synthesis of Au nanoparticles. In this synthesis, a batch of 10 mL 0.25 mM HAuCl_4_ is commonly used to produce about 1.7 × 10^14^ Au nanoparticles. To fabricate the same amount of Au nanoparticles with the template method, a working electrode surface of approximately 0.6 m^2^ is required.

## 5 Conclusions

In this work, we have fabricated ordered arrays of Au nanoparticles with diameters of 25 nm and 46 nm, using PAA as a template and PED as the electrodeposition method. We showed that the aspect ratio of the nanostructures (and thus of their optical resonance properties) can be tuned by simply choosing an appropriate the deposition time. The deposited metal is pure Au (as confirmed by EDS) with a polycrystalline structure. A horizontal grain size of 22 nm and a vertical grain size of 237 nm, in the template with pore diameter of 25 nm, was found. In the template with pore diameter of 46 nm, the horizontal grain size was 36 nm and the vertical grain size was 82 nm. The GIXRD data showed compressive strain in the direction of confinement and expansive strain in the pore direction. In contrast with some traditional colloidal nanocatalysts, the Au nanostructures produced electrochemically in this work are surfactant-free and do not need any additional step involving the removal of capping agents. In future investigations, the fabricated nanostructures could be employed in catalysis and electrocatalysis studies.

## Conflicts of interest

There are no conflicts to declare.

## Supplementary Material
